# Non-circularly shaped conical diffraction

**DOI:** 10.1038/s41598-022-10749-0

**Published:** 2022-05-05

**Authors:** Muhammad Waqar Iqbal, Nicolas Marsal, Germano Montemezzani

**Affiliations:** 1 Université de Lorraine, CentraleSupélec, LMOPS, 57000 Metz, France; 2 Chair in Photonics, CentraleSupélec, LMOPS, 57000 Metz, France

**Keywords:** Optical techniques, Optical manipulation and tweezers

## Abstract

Waves with tailored shape and vectorial non-homogeneous polarization are of much interest due to the many prospects for relevant applications in the classical and quantum domains. Such vector beams can be generated naturally via conical diffraction in optically biaxial crystals. The recent strongly revived attention to this phenomenon is motivated by modern applications such as optical trapping, polarimetry or super-resolution imaging, partly enabled by new configurations increasing the beam complexity, like those with several crystals in cascade. However, up to now all beams generated by conical diffraction conserve at their sharpest plane the underlying circular shape connected with the planar section of light cones. Here we show that a proper manipulation in wave-vector space within a conical diffraction cascade produces vector beams with highly peculiar non-circular forms, leading to an interesting and reconfigurable platform for easily shaping all structured wave properties, increasing complexity and information content. The experimental observations are confirmed by numerical integration of a paraxial model incorporating the effects of the wave-vector space manipulation.

## Introduction

The shaping and structuring of light beams in terms of intensity, phase and polarization distribution is presently attracting wide attention with applications extending from 3D micro-manipulation and imaging, to classical and quantum communication^1,2^. Among the most important custom light fields one counts vector beams that possess a spatially varying light polarization across the beam, as well as beam exhibiting phase singularities and an optical angular momentum (OAM), such as optical vortices. Besides their strong fundamental interest, such tailored light fields are emerging for an increasing number of applications. These include optical manipulation or tweezing^3,4^, the exploiting of OAM for mode division multiplexing in optical communication^5^ or for quantum information^6,7^, super-resolution imaging^8^, as well as other key enabling technologies in fields including metrology, optical machining, biomedicine, chemistry, and several others^1,9^.

Internal conical diffraction (CD, also called internal conical refraction) is a natural phenomenon leading to vector beams of circular shape possessing a fractional OAM. This peculiar effect has intrigued the scientific community since its prediction by W. R. Hamilton nearly 200 years ago and the first experimental observation by H. Lloyd soon after. The CD phenomenon is observed when a sufficiently tightly focused beam is incident on an optically biaxial crystal (BC) with its wave-vector $\mathbf {k}$ parallel to one of the optical axes of the crystal^10^. For this singular $\mathbf {k}$-direction, the Poynting vector directions are degenerate and lie on the surface of a slanted cone with circular base inside the crystal. The section of this cone can be easily visualized in the plane of tighter focusing of the incident wave (focal image plane, FIP), where one observes two closely spaced circularly shaped bright rings (double rings) separated by a dark ring (called Poggendorff ring). Two diametrically opposite points always possess orthogonal linear polarizations, leading to the vector beam character^10–12^. An elegant geometrical explanation on how the Poynting vector directions on the cone depend on the local electric ($\mathbf {E}$) and electric displacement vectors ($\mathbf {D}$) of the wave is given in Born and Wolf’s book^13^. In the last two decades research on CD has been strongly relaunched. This is due on one hand to an improved theoretical understanding of the phenomenon following its paraxial diffraction theory^11^ and on the other hand to its potential for several modern photonics applications in the framework of structured light^10^ among which one may cite optical trapping^14,15^, beam shaping^16,17^, free-space multiplexing for communication^18^, polarimetry^19–21^, super-resolution imaging^22^ or OAM management^23–25^.

A major recent development in the field of CD consists in the study of cascaded configurations, where two or more crystals are put in series with their optical axes being aligned^26–29^. Such a cascaded CD leads to a multiplication of the number of observed rings in the FIP, for *N* crystals one gets $2^{N-1}$ double rings^26^. The cascade still has as free parameters the relative rotation angles $\gamma _n$ (around the common optical axis) of the *n*th crystal with respect to the first in the cascade. For instance, for $N=2$ the relative intensities in the two double rings depend on this angle^29^, for $\gamma _2=0$ (parallel crystals) only the external of the two double rings survives, while for $\gamma _2=\pi$ (antiparallel crystals) only the internal one survives. For a single crystal the radius of the CD double ring corresponds to the product $\mathcal {R}=\alpha L$ of the half-angle of aperture $\alpha$ of the CD cone (depending on the material birefringence) and the crystal length *L*. Importantly, for two BC in cascade of the same kind (same $\alpha$) of lengths $L_1$ and $L_2$ the external and internal double rings have a radius $\mathcal {R}_{ext} \propto L_1+L_2$ and $\mathcal {R}_{int} \propto |L_1-L_2|$, respectively^26,29^. Obviously, if the lengths of the two crystals are identical, the internal double-ring will degenerate into a central spot. There is however an elegant way introduced recently by Peet^30^ to modify these radii by a technique called variable two-crystals cascade. A spherical lens is intercalated between the two cascaded crystals in such a way as to image the FIP near the first crystal into a second FIP near the second BC. It was shown that the magnification factor *M* of this imaging modifies the relative values of the observed double ring radii according to $\mathcal {R}_{ext} \propto M L_1+L_2$ and $\mathcal {R}_{int} \propto |M L_1-L_2|$^30^. This make the effect much more versatile and allows the continuous tuning of the actual cascade parameters despite using crystals of fixed lengths.

With respect to the intensity distribution, if the input wave has circular polarization the intensity along the rings is homogeneous. An inhomogeneous azimuthal distribution can be achieved by linearly polarizing the light at the input or output^10^ or, more drastically, by scrambling the polarization between two or more BCs in cascade^17^, in which case also polarization patterns of increased complexity are obtained. Nevertheless, despite for all the above described developments, the CD patterns obtained so far always possess an intrinsic circular shape. This form is associated to the section of the CD cone with a plane perpendicular to the central light wavevector and the optical axis of the BCs. In this work we show that the circular symmetry can be dramatically broken by a proper manipulation in the wave-vector space between the BCs in a cascade. This leads to CD patterns with highly increased complexity, both in terms of the shapes and of the polarization distribution. The resulting vector beams can be modified in an extremely versatile way by changing the positions of the elements inserted in the cascade that will be described below. Our unexpected experimental observations are shown to be in very good agreement with the predictions of a Fourier domain theoretical model based on paraxial diffraction theory that takes into account the applied wave-vector manipulation.

## Principle, optical set-up and modeling

**Figure 1:**
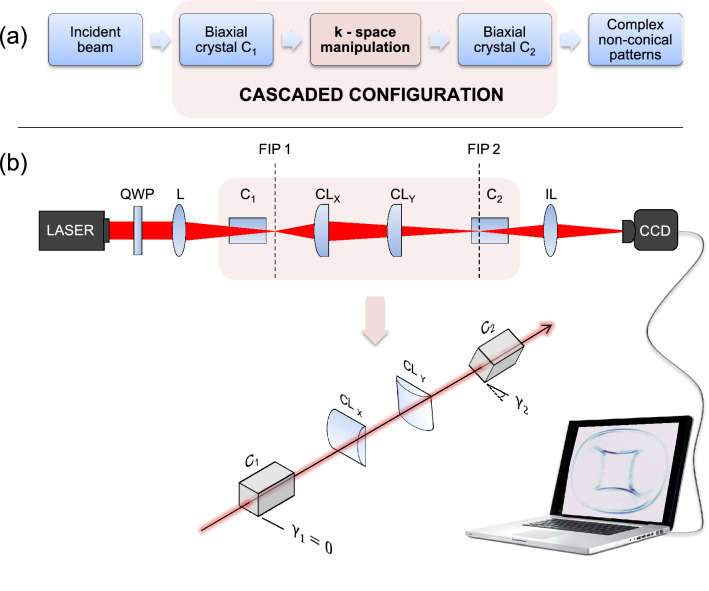
(**a**) Schematic concept for generating non-circularly shaped conical diffraction beams. (**b**) Optical setup; QWP: quarter-wave plate, L: focusing lens, C$_1$/C$_2$: biaxial crystals, CL$_x$/CL$_y$: crossed cylindrical lenses, FIP1/FIP2: focal image planes, IL: imaging lens of FIP2 onto CCD camera. The angle $\gamma _2$ is the rotation angle of crystal C$_2$ with respect to $C_1$ around the common optical axis.

The concept underlying our approach is illustrated in Fig. 1a. The key aspect is in the manipulation of the transverse wave-vector space (*k*-space) that effectively splits this 2D space into two 1D spaces. As seen in the experimental set-up of Fig. 1b, this manipulation is done with the help of two crossed cylindrical lenses CL$_x$ and CL$_y$. The linear polarized He–Ne laser beam (wavelength $\lambda =633$ nm) is transformed to circular polarization by the quarter-wave plate (QWP) and focused by the spherical lens L (focal length $f = 200$ mm) to the first BC (C$_1$). The CD in this crystal can be observed as a sharp round and azimuthally homogeneous double ring at the first focal image plane (FIP1). Importantly, both cylindrical lenses (CL) image this plane into a same plane (the second focal image FIP2) near the position of the second BC (C$_2$), however with different magnification factors $M_x$ and $M_y$, respectively. The choice of identical focal lengths for the two CL would leave only limited versatility for the selection of the $M_x$ and $M_y$ values and it is better to choose different focal lengths, in our case $f_x = 100$ mm for CL$_x$ and $f_y = 75$ mm for CL$_y$. The cascaded CD pattern is formed in FIP2 and is imaged by the imaging spherical lens IL ($f = 100$ mm) on the CCD camera. In our experiments KGd(WO_4_)_2_ is chosen for both BCs with lengths $L_1=22.6$ mm for C$_1$ and $L_2=17.6$ mm for C$_2$. The angles $\gamma _1$ (chosen = 0) and $\gamma _2$ give the relative orientation of the two BCs around the common optical axis, as shown in Fig. 1b.

Before discussing the experimental results, we present briefly the main features of the theoretical model utilized to numerically calculate the expected cascaded CD patterns. The model builds on Berry’s paraxial theory for cascaded CD^26^ by including the effects of the *k*-space manipulation. It is useful to use normalized coordinates in both real and wave-vector space so that the polar coordinate radius *r* in real space becomes $\rho \equiv r/w$, and the small transverse wave-vector $k_t$ in *k*-space (with respect to the exact optical axis direction) becomes $\kappa \equiv k_t w$, with *w* being the 1/e intensity width of the input wave at its focal point^17,26^. We consider an incident paraxial wave field, whose Fourier transform of the electric displacement vector at the position of FIP1 is $\mathbf {D}_0(\kappa , \phi )$ with $\phi$ being the azimuthal angle in polar coordinates. The distribution of the $\mathbf {D}$-field in real space after going through the whole cascade is obtained as

1 $$\begin{aligned} \mathbf {D}(\rho , \varphi ) = \frac{1}{2\pi } \ \int _{0}^{2\pi } \int _{0}^\infty e^{\iota \kappa \rho \cos (\phi - \varphi )} \mathbf{U _{\hbox {tot}}}\mathbf {D}_0(\kappa , \phi ) \kappa \ d\kappa \ d\phi , \end{aligned}$$

where $\varphi$ is the azimuthal angle in real space. Note that here the above real space $\mathbf {D}$-field is calculated at the plane FIP1 at which the input field $\mathbf {D}_0$ and the width *w* are defined. It corresponds to the field at FIP2 back imaged to the plane FIP1 by the CL pair. Therefore a forward imaging of this field to plane FIP2 must be performed in order to compare with the experimental images obtained at the CCD camera. The key element in the above integral is the transfer matrix $\mathbf{U _{\hbox {tot}}}$ that contains the effect of the cascade on each plane wave component composing the input beam, i.e. the effects of the two BCs and of all the optical elements in the path. For our situation this transfer function can be expressed as

2 $$\begin{aligned} \mathbf{U _{\hbox {tot}}}(\kappa , \phi ) = \mathbf{U _{2}}(\kappa ' , \phi ' , \rho _2,\gamma _2) \cdot \ \mathbf{U _{1}}(\kappa , \phi , \rho _1, \gamma _1), \end{aligned}$$

where the matrices $\mathbf{U} _i$ (*i* = 1, 2) are given as

3 $$\begin{aligned} \mathbf{U} _i(\kappa , \phi , \rho _i, \gamma _i) = \exp \left( -\iota \kappa \rho _i \begin{bmatrix} \cos (\phi - \gamma _i) &{} \sin (\phi - \gamma _i) \\ \sin (\phi - \gamma _i) &{} -\cos (\phi - \gamma _i) \end{bmatrix}\right) , \end{aligned}$$

with $\rho _i \equiv \alpha L_i / w$ being the normalized strength parameters for the CD in the two crystals, proportional to their lengths $L_i$. The essential element in Eq. (2) is the transformation in the wave-vector space coordinates from $(\kappa , \phi )$ to $(\kappa ', \phi ')$ between the two transfer matrices **U**$_1$ and **U**$_2$, which reflects the applied *k*-space manipulation. It can be easily shown that the needed transformations are

4 $$\begin{aligned} \kappa ' = \kappa \sqrt{\frac{\cos ^2 \phi }{M_x^2}+\frac{\sin ^2 \phi }{M_y^2}} \end{aligned}$$

and

5 $$\begin{aligned} \phi ' = \arctan \left[ \frac{M_x}{M_y} \left( \frac{-\sin \phi }{-\cos \phi } \right) \right] . \end{aligned}$$

In the latter case one needs to take care of the correct quadrant for the arctangent function in accordance with the signs of numerator and denominator inside the round brackets. The calculations that will be shown below for the two-crystal cascade case are obtained by numerical integration of Eqs. (1) and (2) with an input electric displacement field $\mathbf {D}_0=1/\sqrt{2} \exp (-\kappa ^2/2)(1,i)^T$ corresponding to a circularly polarized gaussian beam. The used strength parameters $\rho _1=17.9$ and $\rho _2=13.9$ reflect the experimental focusing conditions and the crystal lengths. The obtained real space complex output field $\mathbf {D}$ is forward imaged to the plane FIP2 and its local relative intensity is calculated by its square module ($I \propto |\mathbf {D}|^2$).

**Figure 2:**
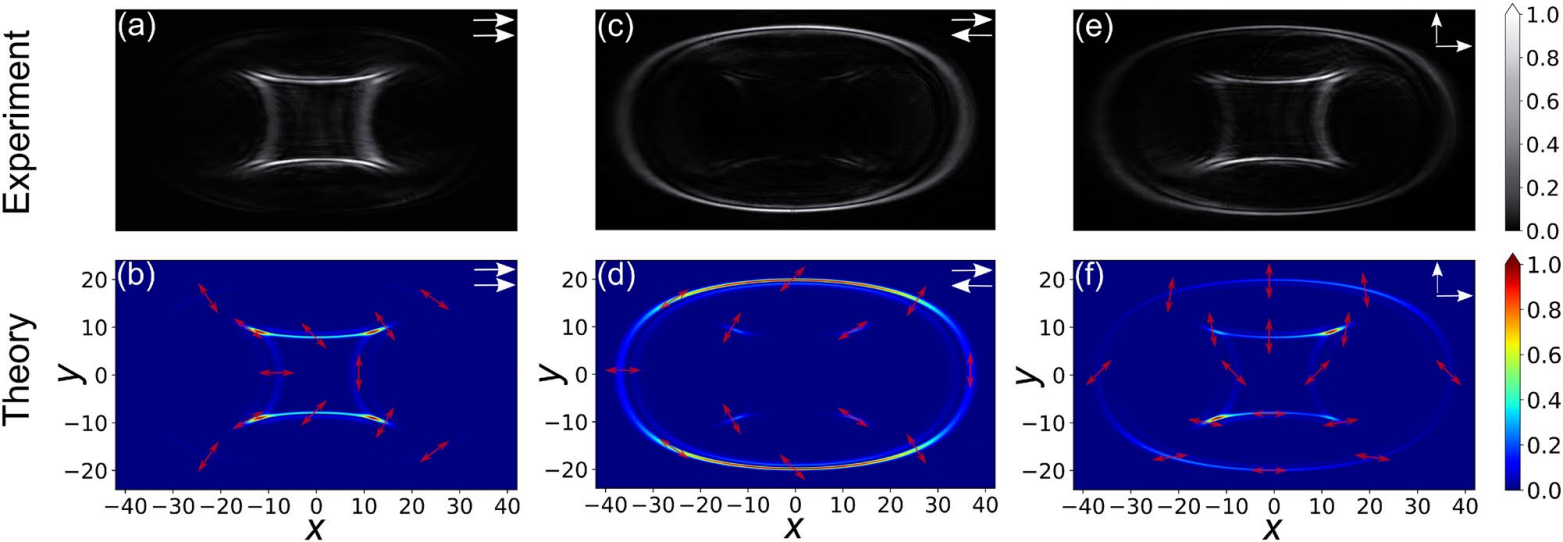
Experimentally observed (upper row) and theoretically calculated (lower row) non-circular conical diffraction patterns for the cases of parallel, (**a**) + (**b**), antiparallel, (**c**) + (**d**), and crossed biaxial crystals, (**e**) + (**f**). The magnifications between the planes FIP1 and FIP2 provided by the crossed CL are $M_x=1.25$ and $M_y=0.325$. The coordinates $x=-M_x \rho \cos \varphi$ and $y=-M_y \rho \sin \varphi$ in the theoretical plots are proportional to the linear dimensions on the CCD camera. The red arrows give the local linear polarization direction. The intensity scales are shown on the right.

## Results and discussion

We consider first the case where the two BCs are either parallel ($\gamma _1=\gamma _2=0$) or antiparallel ($\gamma _2=\pi$), the results are shown in the first two columns of Fig. 2 for magnifications $M_x=1.25$ and $M_y=0.325$. Figure 2a (experiments) and b (theory) show the parallel case. Clearly the pattern is dominated by an internal structure of strongly non-circular shape looking rather like a rotated diamonds symbol in playing cards. This structure has a reversed curvature, it is convex rather than concave if looked from inside. A very faint external structure can also be recognized both in experiments and theory. For the antiparallel case (Fig. 2c,d) the situation is reversed. Here the external structure dominates with only spurious presence of the internal one. This oval external structure is always concave and importantly, cannot be described by the equation of an ellipse, which would be expected by the deformed imaging of a circle with our optical system. Moving radially, both internal and external structures present two intensity maxima separated by a Poggendorff-like zero-intensity region. In conventional and cascaded CD without *k*-space manipulation, the external of each double ring is the most intense. This remains true for the oval outer structure with the stronger features on the outside. However, the internal convex structure presents the most intense features on the inside, which is again an opposite behavior. Obviously in our case the internal structure takes the role of the internal double ring in normal cascaded CD and the external the one of the external double ring. However, besides for the form, two other major differences exist. The first is that for normal cascaded CD only the external double ring is present for the parallel case and vice versa, which is exactly contrary to the present situation. The reason is the switch of the transverse wave-vector components signs at the CLs. The second difference is the fact that in the present case, unlike for normal cascaded CD, the second structure, though very faint, does not vanish completely. This is due to the fact that the destructive interference leading to this disappearance is not fully complete for our anisotropic imaging case. We can also notice that the light polarization on the pattern is always linear but has a complex distribution (see red arrows in theoretical pictures). Any two points connected by a central inversion possess orthogonal polarizations. We also note that, unlike in the case where the patterns would be circular, the intensity distribution within the pattern is highly inhomogeneous. Nevertheless, a symmetry with respect to a horizontal and a vertical axis through the center is present throughout for the cases of Fig. 2a–d. However, this symmetry is lost when the second BC is no longer parallel or antiparallel to the first one, as seen in Fig. 2e,f, where $\gamma _2=\pi /2$. Clearly both structures become roughly equally important and the maximum of intensities are shifted towards one of the diagonals. We attribute this shift to the artificial chirality introduced into the system by the rotation of the second BC. A comparison of the local polarizations in Fig. 2b,d,f shows the crucial role of the angle $\gamma _2$ on the polarizations.

**Figure 3:**
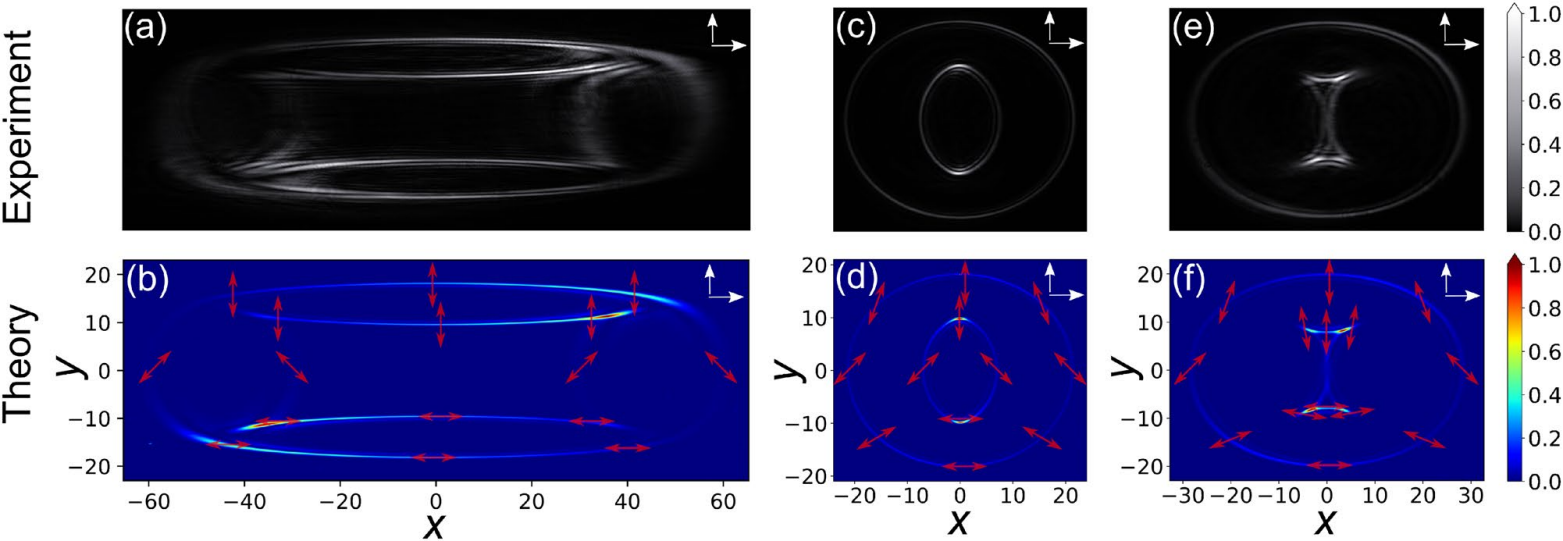
Experimentally observed (upper row) and theoretically calculated (lower row) non-circular CD patterns for crossed crystals ($\gamma _2=\pi /2$) and various combinations of the magnification factors $M_x$ and $M_y$. (**a**) + **(b)**: $M_x=2.50$, $M_y=0.233$; (**c**) + (**d**): $M_x=0.400$, $M_y=0.232$; (**e**) + (**f**): $M_x=0.778$, $M_y=0.323$ (partially degenerate case). Remaining parameters and symbols as for Fig. 2.

The shapes of the CD structures can be easily modified by changing the *k*-space manipulation between the BCs and some examples are given in Fig. 3. The first column (Fig. 3a,b) displays a situation more strongly stretched in *x*-direction (larger $M_x$ and smaller $M_y$). Here the internal and external structures basically merge to give an upper region with essentially vertical polarization and a lower one with nearly horizontal polarization, the two being joint by two weak “rings” with polarizations near $\pm 45^\circ$. The central column (Fig. 3c,d) corresponds to a case where the anisotropic imaging squeezes both directions. Here the pattern is more gentle and all structures are concave, as is the case (not shown) of a stretching in both directions. The light distribution is still strongly inhomogeneous and the lack of symmetry with respect to the horizontal and vertical axes are less evident, but still present.

In our calculations (bottom rows of Figs. 2 and 3) the *x* and *y* axes are scaled as $x=-M_x \rho \cos \varphi$ and $y=-M_y \rho \sin \varphi$, what takes into account the imaging between the calculated pattern at FIP1 and the observed one at FIP2. The positions of the points of intersection of the obtained structures with these axes merit a discussion. Let’s call $\pm X_{\pm }$ and $\pm Y_{\pm }$ the four intersections of the external (subscript +) and internal structure (subscript −) with the *x*-axis and the *y*-axis, respectively. It can be easily verified that these intersection points satisfy well the relations

6 $$\begin{aligned} X_{\pm }=M_x \rho _1 \pm \rho _2; \; Y_{\pm }=M_y \rho _1 \pm \rho _2. \end{aligned}$$

For instance for the case in Fig. 2 these expressions give $X_+ = 36.3$, $X_- = 8.5$, $Y_+ = 19.7$ and $Y_- = -8.1$ in accordance with the numerical calculations. Similarly, for the case of Fig. 3c,d one has $X_+ = 21.1$, $X_- = -6.0$, $Y_+ = 18.1$ and $Y_- = -9.7$. In general the most striking patterns are obtained when either $X_-$ or $Y_-$ is negative, but not both. It is worth noting that the above expressions (6) for $X_{\pm }$ and $Y_{\pm }$ are a generalization of the one given by Peet^30^ for the radii of the double rings in variable two-crystal cascade by intercalation of a spherical lens. Unfortunately our formulas hold only for the intersection points on the main axes and such simple expressions cannot be given for points lying obliquely on the structures.

Following the above argumentation it is interesting to investigate the case where either $X_-=0$ or $Y_-=0$, but not both. This is a partially 1D degenerate situation (degeneration only along one of the two axes) and is a unique feature possible with our *k*-space manipulation that treats the two 1D transverse spaces separately. The 1D degeneration is obtained if $M_x F=1$ or $M_y F=1$, with the factor *F* defined as $F \equiv \rho _1/ \rho _2$. In our experiments $F=L_1/L_2 = 1.284$ and a corresponding 1D degenerated case (along *x*) is shown in Fig. 3e,f. The obtained CD pattern is composed of an external oval and of a complex internal structure resembling a calligraphic letter ${\mathcal {I}}$. Unlike for the other patterns in Fig. 3 that exhibit always linear polarization everywhere, here in the very center of the internal structure components of different polarizations and phases collide and interfere, leading locally to a significant degree of ellipticity. However, away from the degenerate zone the local polarization is still linear also in this exemple.

**Figure 4:**
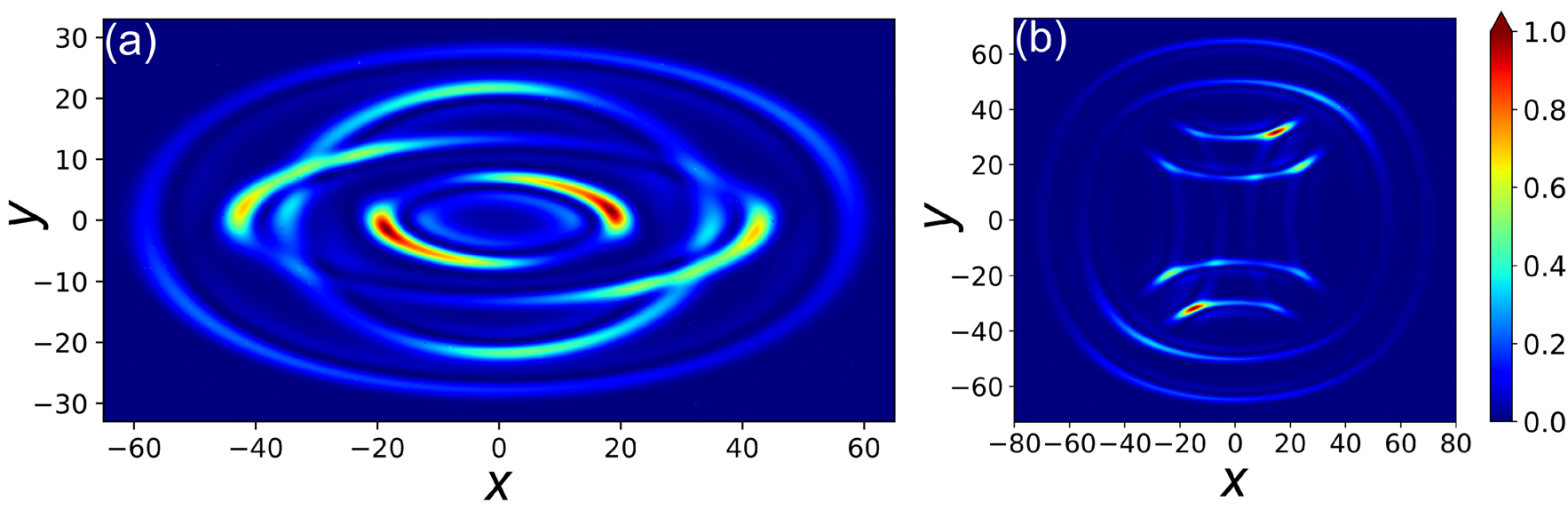
Calculated non-circular CD patterns for a cascade of three mutually crossed crystals ($\gamma _1=0$, $\gamma _2=\pi /2$, $\gamma _3=\pi$) with CD strengths $\rho _1=12.0$, $\rho _2=9.3$ and $\rho _3=7.3$. The magnification factors associated to the *k*-space manipulations between each pair of BCs (see text) are: (**a**) $M_{x1}=2.50$, $M_{y1}=4.30$, $M_{x2}=1.25$, $M_{y2}=0.325$; (**b**) $M_{x1}=1.25$, $M_{y1}=0.325$, $M_{x2}=2.50$, $M_{y2}=4.30$. Therefore the manipulation in (**b**) between the second and third BC is the same as the one in (**a**) between the first and second BC, and vice versa. The color scale gives the relative intensity.

The technique is obviously not limited to the case of two crystals specifically discussed above and the cascade can be extended to *N* crystals with intermediate manipulations in *k*-space. Similar to the case of a conventional *N*-crystals cascade, CD patterns formed by a total of $2^{N-1}$ structures can be expected in this case. Two calculated examples for the case of a cascade of three mutually crossed crystals with wave-vector space manipulation between each pair of them are shown in Fig. 4. The simulations are performed using Eq. (1) and the proper generalization of Eqs. (2), (4) and (5). Here the CD strength parameters of the three BCs are chosen to be $\rho _1=12.0$, $\rho _2=9.3$ and $\rho _3=7.3$ with nearly equal ratios $F_{12} \approx F_{23}$ with $F_{ij} \equiv \rho _i/ \rho _j$. A set of two crossed cylindrical lenses is assumed to be placed between each pair of BCs giving magnifications $M_{x1}$ and $M_{y1}$ between the focal image planes FIP1 and FIP2 (near the first and second crystal), and magnifications $M_{x2}$ and $M_{y2}$ between FIP2 and a third focal image plane FIP3 near the last crystal in the cascade. The calculated intensity images depicted on Fig. 4 are those observable at FIP3. The case of Fig. 4a showing four structures looking like orbits with two of them intersecting each other is obtained for $M_{x1}=2.50$, $M_{y1}=4.30$ and $M_{x2}=1.25$, $M_{y2}=0.325$. Remarkably, the mere permutation of the $M_{x1}$ and $M_{y1}$ values with the $M_{x2}$ and $M_{y2}$ ones leads to a completely different beam shape. This situation is shown in Fig. 4b, where one observes an internal Maltese cross-like shape formed by the two intersecting convex internal structures, combined with two concave external ones. The above permutation of the magnification values corresponds in practice to exchanging the cylindrical lenses between the first and second BC with those between the second and the third. The fact that this operation leads to fully different patterns is related to the lack of commutation of the matrices within the integral (1) associated to a generalization of Eq. (2). Similarly to the relationships (6), the eight intersection points $\pm X_{\pm \pm }$ and $\pm Y_{\pm \pm }$ of the four structures with the *x*- and *y*-axes are found here by

7 $$\begin{aligned} X_{\pm \pm } = M_{x2}(M_{x1} \rho _1 \pm \rho _2) \pm \rho _3 \end{aligned}$$

and

8 $$\begin{aligned} Y_{\pm \pm } = M_{y2}(M_{y1} \rho _1 \pm \rho _2) \pm \rho _3. \end{aligned}$$

The generalization of these expressions to a longer cascade of *N* crystals is straightforward. As it can be easily verified, it is worth noting that the ordering and the signs of the eight values $X_{++}$, $X_{+-}$, $X_{-+}$, $X_{--}$, $Y_{++}$, $Y_{+-}$, $Y_{-+}$ and $Y_{--}$ give a direct qualitative guidance on the kind of structure that can be expected for a given set of parameters.

## Conclusion

We have shown that a proper wave-vector space manipulation within a CD cascade leads to highly structured vector beams with striking non-circular shapes. Our experiments performed with a cascade of two biaxial crystals agree very well with the predictions of a corresponding paraxial diffraction model. The generalization to a longer cascade is straightforward and leads to a further increase in the CD pattern complexity. It is also worth mentioning that the addition of intermediate polarization scrambling by electro-optic elements can lead to a switching of the sub-structures in a way similar to the one proposed in an earlier work^17^, with speeds potentially of the order of GHz. The fast commutation of such highly complex structures is therefore feasible, which may be used also to address resonant phenomena, for instance in connection with optical trapping. Furthermore, the structured beams obtained by the technique described here may lead to multi-chamber and dynamically reconfigurable bottle beams, as an extension to the single chamber bottle beams already demonstrated earlier with CD^31^. Therefore, we believe that the richness and complexity of these naturally created vector beams and the versatility to tailor their properties by just a few parameters allows for a new platform for fundamental and applied studies of structured light. Finally, it is worth mentioning that CD beams present generally very interesting features in terms of the OAM of light^23,24,32^. As cylindrical lenses are known to modify the OAM, we expect the present technique to highly enrich the opportunities for its tailoring and its manipulation.

## Data Availability

The datasets generated and/or analysed during the current study are available from the corresponding author on reasonable request.
